# Ptomain Poisoning by Pork

**Published:** 1897-08

**Authors:** 


**Ptomain Poisoning by Pork.**—Dr. Winter Blyth reports, in the *London Sanitary Chronicle*, the cases of a boy and a girl, aged respectively 11 and 5 years, who were brought to death's door by the ingestion of boiled pork, on a Saturday (March 29).

Forty-eight hours later, the father, the boy and the girl were seized with choleraic symptoms, and at 2:30 Monday afternoon the mother became similarly affected, but in a lesser degree. At 11 a. m. of that day, the boy and the girl had high temperature, the father choleraic symptoms, cramp, numbness of the feet and hands, and smarting of the eyes. The father, the mother and the boy got better by about Tuesday, but the girl by Wednesday morning was almost pulseless, with cold extremities, and very nearly died. She, however, revived, and ultimately recovered. The pork was examined by the medical officer of health Wednesday, April 1, that is, four days after it was bought, three days after it had been boiled. At that date its appearance and odor were perfectly normal, but on passing it through a small sausage machine the finely divided fiber had a distinct stale smell. The pork was analyzed by Gautier's process, and ultimately a crystalline substance not in normal pork was separated. This substance was alkaline in reaction, gave fine crystals when united with hydrochloric acid, and precipitates with the ordinary alkaloidal reagents (save the chloride of gold or platinum). So small a quantity was, however, obtained, that it was not practicable to examine it farther or to determine whether the substance was really a poisonous "ptomain," although this is naturally the inference. It may be suggested that the ptomain was in quantity in the meat on Sunday, and what the writer found was a small residue which had escaped destruction in the process of putrefaction.—*Journal of the American Medical Association.*

